# Nanopartikel auf subnanometer dünnen oxidischen Filmen: Skalierung von Modellsystemen

**DOI:** 10.1002/ange.202015138

**Published:** 2021-01-28

**Authors:** Kevin Ament, Nicolas Köwitsch, Dianwei Hou, Thomas Götsch, Jutta Kröhnert, Christopher J. Heard, Annette Trunschke, Thomas Lunkenbein, Marc Armbrüster, Josef Breu

**Affiliations:** ^1^ Bavarian Polymer Institute and Department of Chemistry University of Bayreuth Universitätsstraße 30 95447 Bayreuth Deutschland; ^2^ Faculty of Natural Sciences Institute of Chemistry Materials for Innovative Energy Concepts Chemnitz University of Technology Straße der Nationen 62 09111 Chemnitz Deutschland; ^3^ Department of Physical and Macromolecular Chemistry Charles University Hlavova 8 128 00 Prague 2 Czech Republic; ^4^ Department of Inorganic Chemistry Fritz-Haber-Institut der Max-Planck-Gesellschaft Faradayweg 4–6 14195 Berlin Deutschland

**Keywords:** CO-Oxidation, Metall-Träger-Wechselwirkung, Palladium, Schichtsilikat, Ultradünne Schichten

## Abstract

Durch die Abscheidung von ultradünnen Oxidschichten auf atomar‐flachen Metalloberflächen konnte die elektronische Struktur des Metalls und hierdurch dessen katalytische Aktivität beeinflusst werden. Die Skalierung dieser Architekturen für eine technische Nutzbarkeit war bisher aber kaum möglich. Durch die Verwendung einer flüssigkristallinen Phase aus Fluorhectorit‐Nanoschichten, können wir solche Architekturen in skalierbarem Maßstab imitieren. Synthetischer Natriumfluorhectorit (NaHec) quillt spontan und repulsiv in Wasser zu einer nematischen flüssigkristallinen Phase aus individuellen Nanoschichten. Diese tragen eine permanente negative Schichtladung, sodass selbst bei einer Separation von über 60 nm eine parallele Anordnung der Schichten behalten wird. Zwischen diesen Nanoschichten können Palladium‐Nanopartikel mit entgegengesetzter Ladung eingelagert werden, wodurch die nematische Phase kollabiert und separierte Nanopartikel zwischen den Schichten fixiert werden. Die Aktivität zur CO‐Oxidation des so entstandenen Katalysators war höher als *z. B*. die der gleichen Nanopartikel auf konventionellem Al_2_O_3_ oder der externen Oberfläche von NaHec. Durch Röntgenphotoelektronenspektroskopie konnte eine Verschiebung der Pd‐3d‐Elektronen zu höheren Bindungsenergien beobachtet werden, womit die erhöhte Aktivität erklärt werden kann. Berechnungen zeigten, dass mit erhöhter positiver Ladung des Pd die Adsorptionsstärke von CO erniedrigt und damit auch die Vergiftung durch CO vermindert wird.

## Graphical abstract

Palladium‐Nanopartikel wurden teilweise von subnanometerdicken Schichten aus Hectorit bedeckt. Aufgrund elektronischer Wechselwirkungen zwischen den Nanopartikeln und den ultradünnen Schichten waren die Nanopartikel positiv geladen. Dies wiederum erhöhte die Aktivität in der Oxidation von Kohlenmonoxid.

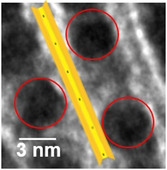

## Einleitung

Eine häufig genutzte Methode zur Generierung von Nanopartikeln auf oxidischen Trägern ist die Imprägnierung mit Präkursoren, gefolgt von chemischer oder thermischer Behandlung. In vielen Fällen wird der oxidische Träger als inert angesehen und dient zur Dispergierung und Stabilisierung der Nanopartikel gegen Ostwald Reifung.^[1]^ Allerdings kann die geschickte Auswahl des richtigen Trägers die Selektivität und Aktivität des nanopartikulären Katalysators signifikant verändern.^[2]^ Es konnte gezeigt werden, dass sogenannte Elektronische‐Metall‐Träger‐Wechselwirkungen (EMSI) Einfluss auf die katalytische Aktivität von Metallen nehmen können.^[3]^ Um diese EMSI‐Effekte zu studieren, wurden in den letzten Jahren Modellsysteme etabliert, die auf ultradünnen oxidischen Schichten auf Metalloberflächen beruhen.[^2a^, ^4^]

Nach Abscheidung des Oxids wurde eine Modifizierung der Austrittsarbeit des Metalls beobachtet.^[5]^ Dieses Phänomen kann verschiedene Ursachen, wie Ladungstransfer, elektrostatische oder Kompressions‐Effekte, haben.[^5b^, ^6^] Für Cluster aus Pt^[7]^ oder Ir^[8]^ auf CeO_2_ Filmen wurde zum Beispiel ein Ladungsfluss vom Edelmetall zum Oxid beobachtet. Dadurch wurde die Metalloberfläche positiv aufgeladen.

Mit Hilfe dieser Modelle konnte ein tieferes Verständnis der Arbeitsweise von Realkatalysatoren geschaffen werden. Zwar können diese Modelle mit höchster Präzision hergestellt werden, jedoch ist die Skalierung von subnanometerdicken Oxidschichten in großem Maßstab nicht praktikabel (Schema 1).^[9]^

**Scheme 1:**
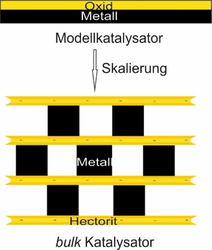
Skalierung der Modellarchitektur einer einzelnen oxidischen Lage (<1 nm) auf einer Metalloberfläche zu einem mesostrukturierten Katalysator mit einer hohen zugänglichen und aktiven Oberfläche. Hierbei wird das Schichtsilikat Hectorit als dünne Oxidschicht verwendet.

Schichtmaterialien mit permanenter negativer Schichtladung, wie Schichtsilikate, sind bekannte Träger für katalytisch aktive Nanopartikel.^[10]^ Durch die Kationenaustauschkapazität der Schichtsilikate können die Zwischenschichtkationen durch Präkursoren ausgetauscht werden, aus denen durch Reduktion (z. B. Pd, Cu, Ru)^[11]^ oder Fällung (CdS)^[12]^ Nanopartikel generiert werden können. Diese Kationenaustauschkapazität von natürlichen Schichtsilikaten liegt in der Größenordnung von <100 mmol/100 g,^[10]^ was die maximal mögliche Beladung limitiert. Im Falle von 3.5 nm großen Pd Nanopartikeln würde dies einer maximal möglichen Beladung von 6 Gewichtsprozent (Gew%) entsprechen, was einer Dichte von einem Nanopartikel pro 1500 nm^2^ Zwischenschichtraum entspricht.

Weiterhin ist die laterale Ausdehnung von natürlichen Schichtsilikaten typischerweise kleiner als 200 nm. Dékány et al.^[13]^ beobachteten, dass Nanopartikel deswegen vor allem auf den äußeren Oberflächen oder Kavitäten erzeugt wurden. Dies spielgelte sich auch in insignifikanten Verschiebungen des Basalflächenabstands wider. Durch die limitierende Kationenaustauschkapazität können höhere Beladungen nur realisiert werden, wenn bereits fertige Nanopartikel interkaliert werden. Bei Zwischenschichtabständen von weniger als einem Nanometer ist dies aus kinetischer Sicht jedoch unwahrscheinlich.

Das synthetische Schichtsilikat Natriumfluorhectorit (NaHec, [Na_0.5_]^inter^[Mg_2.5_Li_0.5_]^oct^[Si_4_]^tet^O_10_F_2_) besitzt die Eigenschaft osmotisch zu quellen.^[14]^ Damit gehört es zu einem kleinen Kreis von Schichtmaterialien, bei denen dieses seltene Phänomen beobachtet werden kann.^[15]^ Es handelt sich um einen thermodynamisch erlaubten Prozess,^[16]^ welcher eine flüssigkristalline Phase erzeugt. Einzelne Schichtsilikatlamellen sind dabei uniform voneinander entfernt. NaHec besitzt eine mittlere laterale Plättchenausdehnung von 20 μm,^[17]^ was es den individuellen Schichten mit einer Dicke von 0.96 nm nicht ermöglicht selbst bei hohen Verdünnungen (<1 Volumenprozent) frei zu rotieren. Dadurch bildet sich eine nematische flüssigkristalline Phase aus.^[18]^ Wie auch für schichtförmige Titanate^[19]^ gezeigt wurde, adaptieren verdünnte Dispersionen von NaHec durch die starke elektrostatische Abstoßung der gleichgeladenen Schichten eine co‐faciale Orientierung. In der nematischen Phase wird diese Geometrie selbst bei großen Abstände zwischen den Schichten (>50 nm) behalten.

Die Verwendung dieser nematischen Phase von NaHec zur Interkalation von Nanopartikeln wurde bereits anhand von Maghemit Nanopartikeln gezeigt.^[20]^ Außerdem bietet die Nutzung einer nematischen Phase die Möglichkeit für eine skalierbare Syntheseroute hin zu nanopartikulären Katalysatoren zwischen subnanometer dicken oxidischen Schichten. Pd Nanopartikel werden zunächst über eine bekannte Synthese^[21]^ hergestellt und mit 4‐Dimethylaminopyridin (DMAP) als Ligand bedeckt. Auf diese Weise entstehen nanopartikuläre, metallische Kationen, die ohne kinetische Hinderung zwischen die separierten Nanoschichten diffundieren können. Dies entspricht im Prinzip einem Kationenaustausch (Schema 2). Der Einfluss der Nanoschichten auf (elektronische) Eigenschaften der Pd Nanopartikel wurde anhand der Oxidation von Kohlenmonoxid (CO) untersucht.

**Scheme 2:**
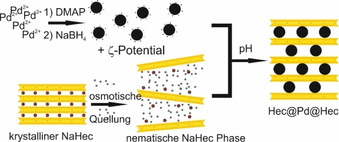
Schema zur Synthese von Pd interkaliertem Hec (Hec@Pd@Hec).

## Ergebnisse und Diskussion

### Synthese und Charakterisierung der Hec@Pd@Hec Katalysatoren

Die synthetisierten Pd Nanopartikel besaßen laut Transmissionselektronenmikroskopie (TEM) eine enge Größenverteilung von 3.5±0.4 nm (Abbildung S1a). Diese waren laut Dynamischer Lichtstreuung (DLS) stabil in Wasser dispergierbar und wiesen einen hydrodynamischen Durchmesser von 4.5±1.3 nm auf. Durch Anpassung des pH‐Wertes konnte das ζ‐Potential zwischen +34 und +14 mV eingestellt werden (Abbildung S1b). Da die Schichtladung und damit die Ladungsdichte von NaHec fix ist, bestimmt die Oberflächenladung der Nanopartikel die Beladung, die nötig für den Ladungsausgleich ist (Tabelle 1). Für die Interkalation wurden die Nanopartikel als 0.1 Gew% Dispersion verwendet und Hectorit wurde als 1.5 Gew% Dispersion unter Rühren zugegeben. Bei diesem Gehalt an NaHec betrug der Abstand zweier Nanoschichten mehr als 60 nm, was durch Kleinwinkelstreuung nachgewiesen wurde (Abbildung S2). Dieser hohe Abstand ermöglichte eine schnelle Diffusion der Nanopartikel zwischen den Schichten. Durch die gegensätzliche Ladung von Nanoschichten und Nanopartikeln wurde eine Heterokoagulation ausgelöst und die Nanopartikel zwischen den Lamellen fixiert. Laut energiedispersiver Röntgenspektroskopie (REM‐EDX) ist das Pd uniform über das Schichtsilikat verteilt (Abbildung S3). Außerdem war laut Elementaranalyse (CHN) DMAP durch wiederholtes Zentrifugieren und Waschen komplett entfernbar (Tabelle S1). Die so hergestellten Katalysatoren werden als Hec@Pdx@Hec bezeichnet, um die sandwichartige Architektur hervorzuheben. X bezeichnet dabei die Beladung in Gew% laut optischer Emissionsspektrometrie mit induktiv gekoppeltem Plasma (ICP‐OES). Die Beladung wurde zudem durch REM‐EDX bestätigt (Tabelle 1). Das Zwischenschicht Na^+^ wurde laut ICP‐OES, REM‐EDX (kein Signal bei 1.04 keV, Abbildung S3) und Röntgenphotoelektronenspektroskopie (XPS, kein Signal für Na 1s bei 1070 eV, Abbildung S4) vollständig entfernt. Dies weist darauf hin, dass der Ladungsausgleich tatsächlich durch die interkalierten Pd Nanopartikel vollzogen wird.

**Table 1:** Gewichtsanteil an Pd abhängig von pH der NaHec‐ und Nanopartikel‐ Dispersion.

Bezeichnung	pH	ζ‐Potential [mV]	Pd‐Beladung (ICP‐OES) [Gew%]	Pd‐Beladung (REM‐EDX) [Gew%]
Hec@Pd65@Hec	9.5	28	65.2	67.8
Hec@Pd72@Hec	10.8	22	72.5	76.0

Wie für einen Quasi‐Ionenaustausch in der Einleitung vermutet wurde, konnte die Beladung an Pd bis zu einem Maximum von 72.5 Gew% (Hec@Pd72@Hec) gesteigert werden. Im Gegensatz zur üblichen Ionenaustausch‐Route können durch Interkalation von positiv geladenen Nanopartikeln sehr hohe Beladungen erzielt werden. Eine Beladung mit 65.2 Gew% (Hec@Pd65@Hec) würde eine Stöchiometrie von Pd_6.7_Mg_2.5_Li_0.5_Si_4_O_10_F_2_ bedeuten. Durch Ionenaustausch von Na^+^ durch Pd^2+^ und anschließender Reduktion wäre die Zusammensetzung auf Pd_0.25_Mg_2.5_Li_0.5_Si_4_O_10_F_2_ limitiert.

**Figure 1:**
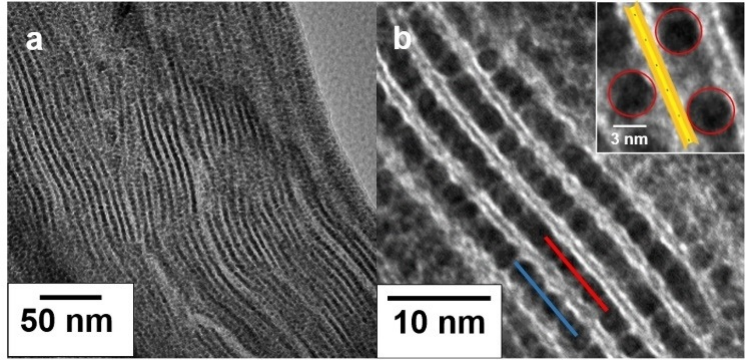
TEM Aufnahmen von Hec@Pd65@Hec bei verschiedenen Vergrößerungen. Die roten und blauen Linien wurden zur Graustufenanalyse (Abbildung S5) verwendet. Der inset zeigt benachbarte Nanopartikel, die voneinander separiert sind.

Durch die Heterokoagulation kollabiert die nematische Phase zu einer lamellaren Struktur, wobei benachbarte Hec Nanoschichten die Nanopartikel fixieren (Abbildung 1). Dabei sind die Nanopartikel nicht dicht gepackt, sondern sind voneinander separiert (Abbildung 1 b, inset). Jede Nanopartikelschicht ist von der nächsten durch exakt eine 0.96 nm dicke Silikatschicht getrennt. Es entsteht eine Struktur wie in Schema 1 beschrieben, wobei die Nanopartikel gegeneinander verschoben sind und gegenüber einem Nanopartikel eine Mesopore gefunden werden kann (Abbildung 1 b, inset). Dies wurde auch durch Graustufenanalyse entlang der blauen und roten Linie in Abbildung 1 b weiter bestätigt (Abbildung S5). Es sollte erwähnt werden, dass die Nanopartikel ihre sphärische Form auch nach der Entfernung des Liganden behalten haben. Längeres Tempern führte jedoch zu einer Verformung (Abbildung S6).

**Figure 2:**
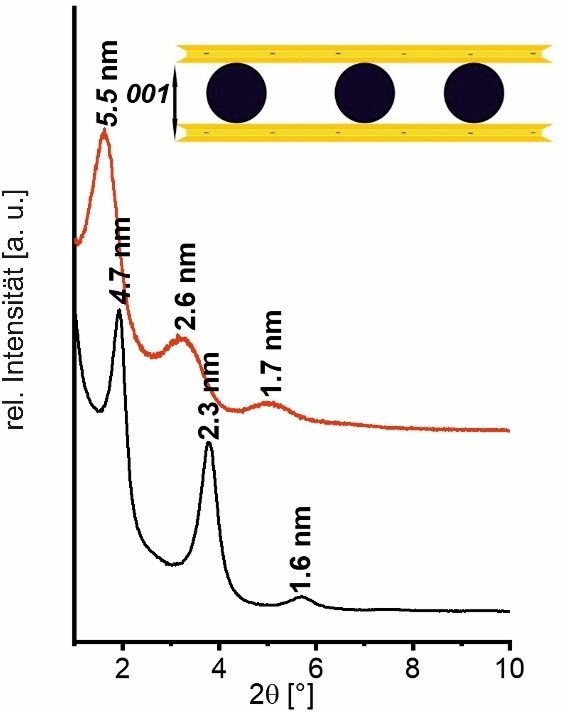
Diffraktogramme von Hec@Pd65@Hec (schwarz) und Hec@Pd72@Hec (rot).

Die monomodalen Pd Nanopartikel erzeugten entlang der Stapelung eine eindimensional periodische Struktur. Die Periodizität betrug laut TEM 4.6±0.7 nm. Bei noch höherer Beladung (Hec@Pd72@Hec) entstanden in einigen Zwischenschichten Multilagen an Pd Nanopartikeln (Abbildung S7), was als Störung der Periodizität angesehen werden kann. Dies geschieht vermutlich, da die Oberflächenladungsdichte bei pH 10.8 (22 mV ζ‐Potential) zu niedrig war, um den Ladungsausgleich der anionischen Nanoschichten in einer Monolage zu erzielen. Pulverdiffraktometrie (PXRD) von texturierten Proben bestätigten die eindimensional kristalline Ordnung. Eine rationale *00l* Serie mit einer Periodizität von 4.7 nm wurde gefunden, die in gutem Einklang mit der TEM‐Bestimmung steht (Abbildung 2). Die Summe aus der Dicke einer Lamelle von 0.96 nm und dem Durchmesser eines Nanopartikels von 3.5 nm würde einen Wert von 4.46 nm ergeben. Bei einer Beladung von 72 Gew% (Hec@Pd72@Hec) führten die Störungen in der Periodizität durch die wenigen Multilagen zu einer statistischen Wechsellagerung und einer Verschiebung der *00l* Serie zu 5.5 nm. Dies wurde von einer Erhöhung der Halbwertsbreite der Reflexe begleitet. Der höhere Schichtabstand lässt sich deswegen mit einer statistischen Wechsellagerung aus Mono‐ und Doppellagen aus Pd Nanopartikeln erklären, da der Röntgenstrahl über die unterschiedlichen Schichtabstände innerhalb seiner Kohärenzlänge mittelt.

Die TEM Aufnahmen ließen bereits vermuten, dass die Nanopartikel in der Zwischenschicht nicht dicht gepackt sind, sondern Poren zwischen den Nanopartikeln existieren müssen. Dies konnte über Ar‐Physisorption und CO‐Chemisorption bestätigt werden (Tabelle 2 und Abbildung S8). Die d_50_ Porenverteilung von Hec@Pd65@Hec beträgt 4.3 nm, was in der Größenordnung eines Pd Nanopartikels liegt. Dies lässt annehmen, dass etwa die Hälfte des Zwischenschichtraums tatsächlich leerer Raum ist. Dies macht auch wahrscheinlich, dass sich gegenüber von einem Nanopartikel auf der anderen Seite der Lamelle eine Mesopore befindet (Schema 1). Zum Vergleich deutete die Ar‐Isotherme von reinem NaHec eine unporöse Struktur mit einer BET Oberfläche von lediglich 4 m^2^ g^−1^ an. Die Schichten sind kollabiert und Ar hat keinen Zugang zur internen Oberfläche von NaHec. Die Struktur wird nur durch Interkalation von Pd Nanopartikeln, die als Säulen fungieren, porös.

**Table 2:** Ergebnisse der Ar‐Physisorption^[a]^ und Chemisorption von CO.^[b]^

Bezeichnung	S_BET_ [m^2^ g^−1^]	Poren‐ größe [nm]	Poren‐ volumen [cc g^−1^]	Metall‐ dispersion [%]
NaHec	4	/	/	/
Hec@Pd65@Hec	147	4.3	0.132	23.7
Hec@Pd72@Hec	87	3.8	0.088	19.9

[a] bestimmt durch Ar‐Physisorption bei 87 K. [b] bestimmt durch CO Doppelisothermen‐Methode.

Die Metalldispersion (Verhältnis von Oberflächen‐ zu Volumenatomen) für einen freien, sphärischen Pd Nanopartikel mit einer Größe von 3.5 nm beträgt 32 %. Durch die gute Zugänglichkeit der Pd Nanopartikel betrug die experimentell bestimmte Dispersion von Hec@Pd65@Hec durch CO‐Chemisorption 24 %. Der Oberflächenverlust kann mit der Bedeckung der Nanopartikel mit den Nanoschichten erklärt werden.

### Untersuchung der katalytischen Aktivität

**Figure 3:**
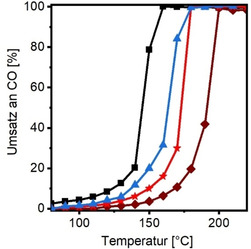
Light‐off Kurven der CO Oxidation: Hec@Pd65@Hec (schwarz), Hec@Pd72@Hec (blau), Pd_ext_@Hec (rot) und Pd_ext_@Al_2_O_3_ (braun). Bedingungen: 50 mL min^−1^ (1 Vol % CO, 1 Vol % O_2_ 98 % N_2_).

Die Oxidation von Kohlenmonoxid (CO) zu Kohlendioxid (CO_2_) wurde als einfache Testreaktion gewählt, um die Aktivität der Katalysatoren in Bezug auf elektronische Metall‐Träger‐Wechselwirkungen resultierend aus der besonderen Architektur zu untersuchen. Aufgrund der hohen Toxizität ist die CO‐Oxidation von enormer Bedeutung für die Abgasnachbehandlung.^[22]^ Für jeden katalytischen Test wurde die Menge an Katalysator so gewählt, dass 1 mg Pd verwendet wurde. Der Gasstrom betrug 50 mL min^−1^ (1 Vol % CO, 1 Vol % O_2_, 98 % N_2_) und light‐off Kurven im Temperaturfenster von 80 °C bis 220 °C wurden bestimmt. Jeder Katalysator wurde dreimal zykliert. Die dritte Kurve ist in Abbildung 3 und Tabelle 3 dargestellt. Alle drei konsekutiven Kurven werden exemplarisch für Hec@Pd65@Hec in Abbildung S9 gezeigt.

**Table 3:** Katalytische Charakterisierung der (Referenz)‐Katalysatoren.

Probe	T_10_ [°C]	T_50_[°C]	T_90_ [°C]	E_A_ [kJ mol^−1^]
Hec@Pd65@Hec	124	145	156	42
Hec@Pd72@Hec	138	163	173	51
Pd_ext_@Hec	150	172	177	49
Pd_ext_@Al_2_O_3_	169	191	198	57

Die Temperatur bei 50 % Umsatz (T_50_) von Hec@Pd65@Hec, welches die Architektur aus Schema 1 am besten widerspiegelt, betrug 145 °C. Die Aktivierungsenergie E_A_ (unterhalb von 10 % Umsatz bestimmt) betrug 42 kJ mol^−1^ (Abbildung S10), was geringer als auf Trägern wie γ‐Al_2_O_3_ oder MgO (55–80 kJ mol^−1^)^[23]^ oder Silica (65–120 kJ mol^−1^)^[24]^ ist.

Die hohe katalytische Aktivität von Hec@Pd65@Hec könnte dabei auf verschiedene Faktoren zurückgeführt werden: Auf die extrem elektronegativen Fluoridanionen im Hectorit; auf die Stabilisierung von atomardispergierten Pd; das mesoporöse Konfinement oder auf elektronische Wechselwirkung zwischen Träger und Metall, wie es bereits in der Einleitung vorgestellt wurde.

Das Fluorid ist nicht an der Oberfläche der Nanoschicht und damit nicht in direktem Kontakt zum Pd, dennoch könnte es Einfluss auf die Aktivität nehmen. Diese Fragestellung kann jedoch nur in Zukunft beantwortet werden, wenn schichtförmige Träger ohne Fluorid verwendet werden.

Zum zweiten Punkt: Die Nanopartikel wurden mehrfach dialysiert vor der Interkalation. Dadurch sollten kleine Cluster entfernt worden sein. Dennoch könnten diese auch durch Auflösung während der Interkalation entstanden sein. Die externen Flächen des Hectorits tragen auch eine negative Ladung und sollte demzufolge in der Lage sein diese Cluster ebenfalls zu stabilisieren. Aus diesem Grund wurden die gleichen Pd Nanopartikel, die auch für Hec@Pd65@Hec verwendet wurden, auf die äußere Oberfläche von nicht gequollenem NaHec abgelagert (Pd_ext_@Hec, Abbildung S11a und Tabelle S2). Die bestimmte T_50_ war jedoch deutlich höher mit 172 °C. Dies weist darauf hin, dass es nicht oder zumindest nicht nur kleine Cluster sein können, die für die hohe Aktivität verantwortlich sind.

Hec@Pd65@Hec und Hec@Pd72@Hec besitzen ähnliche Porengrößen, wobei die Metalldispersion von letzterem niedriger ist. Die katalytische Aktivität von Hec@Pd72@Hec ist dennoch deutlich geringer. Die T_50_ steigt von 145 °C (Hec@Pd65@Hec) auf 163 °C, was bereits nah an Pd_ext_@Hec liegt. Weiterhin stiegen auch E_A_ auf 51 kJ mol^−1^ für Hec@Pd72@Hec bzw. 49 kJ mol^−1^ für Pd_ext_@Hec. Die Verringerung der Aktivität ist deutlich ausgeprägter als es der Unterschied in der Dispersion erwarten lassen würde.

Es scheint, dass der letzte Aspekt, die besondere Architektur tatsächlich eine wichtige Rolle spielt. Aus diesem Grund wurde γ‐Al_2_O_3_ mit einer deutlich geringeren Oberflächenladungsdichte (−20 mV bei pH 10) mit 1 Gew% Pd Nanopartikeln beladen (Pd_ext_@Al_2_O_3_, Abbildung S11b und Tabelle S2). Die T_50_ lag hier bei 191 °C und E_A_ bei 57 kJ mol^−1^ (Abbildung S10), was in guter Übereinstimmung mit der Literatur ist.^[23a]^ Natürlich ist die Oberflächenchemie von Al_2_O_3_ anders als die des Hectorits, dennoch lässt dies vermuten, dass die besondere Architektur zusammen mit der hohen Ladungsdichte des Hectorits (1 negative Ladung pro 48 Å^2^) Einfluss auf die katalytische Aktivität nimmt.

**Figure 4:**
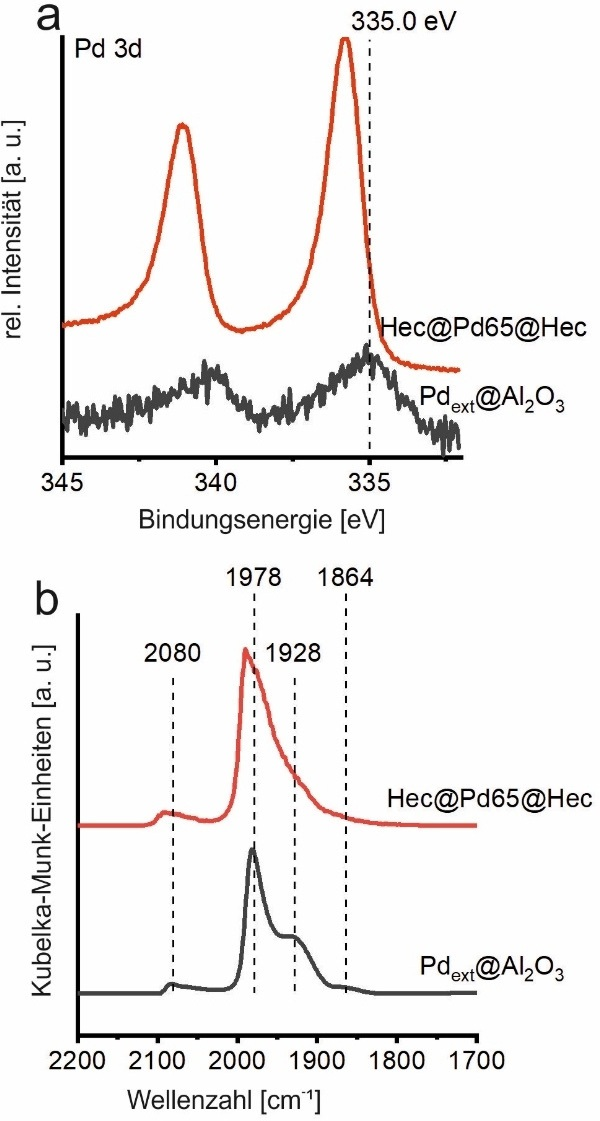
Charakterisierung der Pd Nanopartikel auf Hec@Pd65@Hec (rot) und Pd_ext_@Al_2_O_3_ (schwarz). a) XP Spektren der Pd 3d Region und b) DRIFT Spektren von CO chemisorbiert bei 300 K an die Oberfläche des Pd bei einem Gleichgewichtsdruck von 2 mbar CO.

Wie bereits zuvor erwähnt, müssen die Pd Nanopartikel die permanente negative Ladung der Hec Nanoschichten ausgleichen. XPS der Pd 3d Region von Hec@Pd65@Hec zeigte asymmetrische Signale mit Bindungsenergien (BE) von 335.8 eV für das Pd 3d_5/2_ Signal und 341.0 eV für das Pd_3/2_ Signal (Abbildung 4 a).

Im Vergleich zu bulk Pd Metall (Pd 3d_5/2_ Signal bei 335.0 eV) sind diese zu höheren Werten verschoben.^[25]^ Pd Nanopartikel auf Al_2_O_3_ zeigen Signale im Bereich von 335.0–335.5 eV^[26]^ bzw. 334.8–335.4 eV auf SiO_2_.^[27]^ Die hier beobachtete Verschiebung könnte auf eine elektronendefizitäre Spezies Pd^δ+^ zurück zu führen sein.^[26a]^ Für Hec@Pd72@Hec wurde eine geringere Verschiebung beobachtet (335.5 eV, Abbildung S12), was auch durch das geringere Oberflächenpotential der Nanopartikel bei der Synthese zu erwarten war. Das Pd 3d_5/2_ Signal der Pd Nanopartikel auf der äußeren Oberfläche von NaHec (Pd_ext_@Hec) oder auf γ‐Al_2_O_3_ (Pd_ext_@Al_2_O_3_) waren lediglich zu 335.3 eV bzw. 335.2 eV verschoben (Abbildung 4 a und S12). Dieser Trend zeigt, dass sowohl die negative Schichtladung als auch die besondere Architektur von Hec@Pd@Hec Einfluss auf die elektronische Struktur der Pd Nanopartikel haben. Dieser Trend spiegelt sich auch in der katalytischen Aktivität wider. “Kationisches” Gold wurde ebenfalls als Grund für eine erhöhte katalytische Aktivität in der CO Oxidation identifiziert.^[28]^

Berechnungen mit Hilfe der Dichtefunktionaltheorie (DFT) von Pd auf dem Zeolith FAU gaben Hinweise, dass positiv geladene Pd Atome zu geringeren Aktivierungsbarrieren im Langmuir‐Hinshelwood Mechanismus führen.^[29]^ Die erhöhte Aktivität von Pd^δ+^ wurde mit einer geringeren Bindungsstärke von CO an positiv geladenes Pd erklärt.^[23c]^

Die Adsorption von CO ist sehr empfindlich in Bezug auf die Oberflächenbeschaffenheit von Pd. Eine Veränderung des Pd im Hinblick auf eine mögliche Oberflächenladung kann deshalb in der Veränderung der C‐O Streckschwingung verfolgt werden. Aus diesem Grund wurde Diffuse Reflexions‐Fourier‐Transform‐Infrarotspektroskopie (DRIFTS) an Hec@Pd65@Hec und Pd_ext_@Al_2_O_3_ durchgeführt (Abbildung 4 b). Bei Erhöhung des CO Partialdrucks bis 60 mbar traten ausgeprägte Verschiebungen der Streckschwingung aufgrund von dipolaren Kopplungen durch die steigende Oberflächenbedeckung auf (Abbildung S13). Nach anschließender Einstellung eines Gleichgewichtsdrucks von CO auf 2 mbar konnten für Pd_ext_@Al_2_O_3_ vier Banden bei 2080, 1976, 1928 und 1864 cm^−1^ beobachtet werden (Abbildung 4 b). Diese können verschiedenen Adsorptionsmoden von CO auf der Pd Oberfläche zugewiesen werden. Die Bande bei 2080 cm^−1^ beschreibt die lineare Anbindung von CO an Ecken bzw. Kanten.^[30]^ CO Moleküle, die verbrückend zwischen zwei Pd Atomen auf Stufen der Nanopartikel adsorbieren, können der Bande bei 1978 cm^−1^ zugeordnet werden.^[31]^ Die beiden breiten Banden bei kleinerer Wellenzahl (1928 und 1864 cm^−1^) beschreiben verbrückte bzw. CO Moleküle zwischen drei Pd Atomen auf verschiedenen Kristallebenen.^[31]^ Bei gleichem Gleichgewichtsdruck zeigt das DRIFT Spektrum von Hec@Pd65@Hec ebenfalls vier Banden bei 2094, 1991, 1945 und 1877 cm^−1^. Im Vergleich zu Pd_ext_@Al_2_O_3_ waren diese alle zu höheren Wellenzahlen verschoben (Abbildung 4 b). Bei einer leicht positiv geladenen Oberfläche, wie es für Hec@Pd65@Hec hier vorgeschlagen wird, ist das Vermögen zur π‐Rückbindung vermindert. Durch die verringerte Besetzung der anti‐bindenden 2π* Orbitale des CO wird die interne C‐O Bindung gestärkt, was zu einer Verschiebung der C‐O‐Streckschwingung zu höheren Wellenzahlen führt.^[31a]^ Durch die geschwächte Rückbindung würde dies insgesamt zu einer verringerten Adsorptionsstärke des CO an die Pd Oberfläche führen.

**Figure 5:**
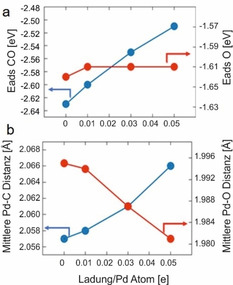
a) Berechnete Adsorptionsenergien bei geringer CO (blau) und O (rot) Bedeckung als Funktion der Nanopartikeloberflächenladung. b) Gemittelte Pd‐C (blau) und Pd‐O (rot) Bindungslängen als Funktion der Nanopartikeloberflächenladung.

Um diese Hypothese weiter zu untermauern, wurde der Einfluss einer positiven Oberflächenladung der Pd Nanopartikel auf die Adsorptionsstärke von CO mit Hilfe der DFT berechnet. Die CO/O Adsorption wurde an dem repräsentativen Modell eines ikosaedrischen Nanopartikels Pd_147_ betrachtet. Dieser hat einen Durchmesser von 1.5 nm und besitzt *(111)* Mikrofacetten, die in sehr guter Näherung eine ausgedehnte *(111)* Oberfläche approximieren. CO und atomarer Sauerstoff wurden auf die *hcp* Besetzungsplätze einer metallischen Oberfläche adsorbiert, wobei ein Bedeckungsgrad θ von 0.1 angenommen wurde (weitere Details des Modells und der Berechnungen sind in der SI zu finden, Abbildung S14–S17). Die Adsorptionsenergie von CO sank linear mit einer Erhöhung der positiven Ladung auf dem Nanopartikel im betrachteten Bereich von Pd_147_ bis Pd_147_^7+^. Dahingegen blieb die Adsorptionsenergie von O unverändert (Abbildung 5). Mit der Verringerung der CO Adsorptionsenergie verlängert sich auch die Pd‐C Bindungslänge von 2.057 Å auf 2.066 Å und die C‐O Bindungslänge verkürzte sich von 1.197 Å auf 1.187 Å. Dies bestätigt, dass eine Verschiebung der Banden der C‐O‐Streckschwingung von einer leicht positiv geladene Pd Oberfläche herrühren kann. Die Verringerung von Elektronen in den Metall d‐Zuständen nahe der Fermi‐Kante führt zu einer verringerten Besetzung des nicht‐bindenden π‐Kanals zwischen CO und Pd. Dadurch wird die Adsorptionsenergie verringert, wobei gleichzeitig die interne C‐O Bindung gestärkt wird^[32]^ Die positive Oberflächenladung könnte die CO Oxidation durch eine gemilderte CO‐Vergiftung der Pd Oberfläche begünstigen. Berechnungen^[29]^ bestätigten zudem, dass die Energiebarrieren für ein positiv geladenes Pd durch die veränderte Bindungsstärke von CO gesenkt werden. Dies steht auch in Einklang mit den verringerten experimentell bestimmten Aktivierungsenergien für Hec@Pd@Hec.

Die permanente und hohe Schichtladungsdichte des Hec ist möglicherweise nicht der einzige Grund für die beobachtete positive Oberflächenladung der interkalierten Pd Nanopartikel. Wie bereits erwähnt, können elektronische Wechselwirkungen zwischen ultradünnen, jedoch neutralen Oxidschichten und Metall auftreten und die Austrittsarbeit des Metalls beeinflussen.[^5^, ^6^]

**Figure 6:**
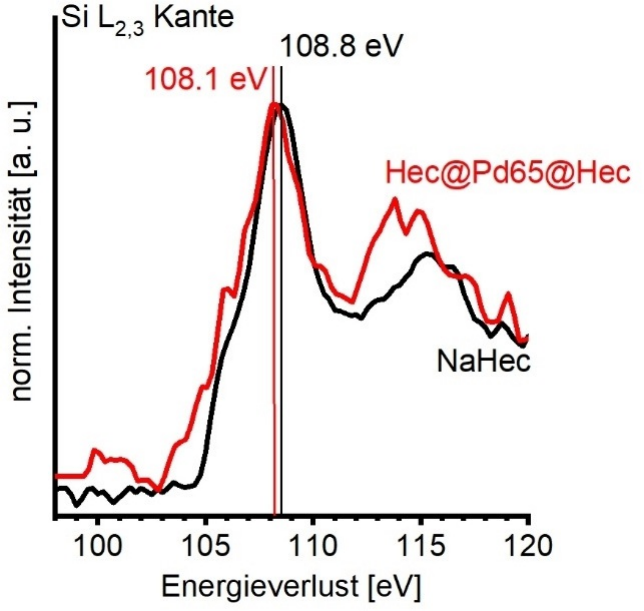
ELL Spektren an der Si L_2,3_ Kante von NaHec (schwarz) und Hec@Pd65@Hec (rot).

Eine ultradünne Schicht aus SiO_2_ auf Mo*(112*) erhöhte beispielsweise die Austrittsarbeit um 0.5–1 eV durch Dipolwechselwirkungen aufgrund von Ladungsübertragung vom Metall auf das Oxid.^[6b]^ Um einen möglichen Ladungstransfer zwischen Pd und Hec zu prüfen, wurde deswegen Elektronenenergieverlustspektroskopie (EELS) an der Si L_2,3_ Kante durchgeführt (Abbildung 6). Im Vergleich zu reinem NaHec trat eine chemische Verschiebung beider Signale bei 109 eV und bei 116 eV zu kleineren Verlusten für Hec@Pd65@Hec auf. Eine Verschiebung von 0.8 eV zu kleineren Verlusten wurde auch an der Si K Kante beobachtet (Abbildung S18). Dies impliziert eine kleine, aber dennoch messbare Reduzierung der mittleren Oxidationsstufe von Si^x^ (*x*< +4) durch die Interkalation der Nanopartikel.^[33]^ Wie bereits an den Modellsystemen[^5^, ^6^] beschrieben und auch wenn das Pd bereits eine positive Ladung aufgrund des Ladungsausgleiches trägt, regt die Bedeckung des Pd mit Hec Nanoschichten einen zusätzlichen Transfer von Ladungsdichte an. Ein Ladungstransfer wurde unter anderem auch von Li et al.^[34]^ gefunden, die Pd Nanowürfel mit dem Cu beinhaltenden MOF HKUST‐1 überwuchsen. Auch hier wurde eine Verschiebung des Pd 3d Signals zu höheren BE durch Ladungstransfer festgestellt. Cu‐O Gruppen des MOF wurden dabei als die Ladungsempfänger festgestellt.^[32a]^

## Schlussfolgerung

Für Modellsysteme, bestehend aus ultradünnen Schichten eines Oxides auf einer Edelmetalloberfläche, konnte gezeigt werden, dass die elektronische Struktur des Metalls beeinflusst wird. Diese Architektur kann auf einer skalierbaren Ebene imitiert werden, indem positiv geladene Nanopartikel zwischen negativ geladene Schichtsilikatlamellen eingeklemmt werden. Wie auch für die Modellsysteme beobachtet, konnten elektronische Wechselwirkungen zwischen Pd und den Nanoschichten mit Hilfe von XPS, EELS und CO‐DRIFTS nachgewiesen werden, die sich auch in einer erhöhten katalytischen Aktivität in der Oxidation von CO widerspiegelten. Die Verwendung einer nematischen Phase eines delaminierbaren Schichtmaterials zur Interkalation von Nanopartikeln ist keineswegs auf Pd oder NaHec beschränkt, sondern auf ein breites Spektrum von Metall‐Nanopartikeln unterschiedlicher Größe und Form anwendbar.^[35]^ Außerdem können andere schichtförmige Träger verwendet werden, die flüssigkristalline Phasen ausbilden, wie zum Beispiel Titanate^[15b]^ und Antimonphosphate.[^15c^, ^15e^] Natürlich lässt sich dieses Konzept auch auf katalytisch attraktivere Legierungen aus Nanopartikeln ausweiten.^[36]^

## Conflict of interest

Die Autoren erklären, dass keine Interessenkonflikte vorliegen.

## Supporting information

As a service to our authors and readers, this journal provides supporting information supplied by the authors. Such materials are peer reviewed and may be re‐organized for online delivery, but are not copy‐edited or typeset. Technical support issues arising from supporting information (other than missing files) should be addressed to the authors.

Supplementary
